# Re-challenge Studies in Non-celiac Gluten Sensitivity: A Systematic Review and Meta-Analysis

**DOI:** 10.3389/fphys.2017.00621

**Published:** 2017-09-05

**Authors:** Elena Lionetti, Alfredo Pulvirenti, Martina Vallorani, Giulia Catassi, Anil K. Verma, Simona Gatti, Carlo Catassi

**Affiliations:** ^1^Department of Pediatrics, Università Politecnica delle Marche Ancona, Italy; ^2^Department of Clinical and Molecular Biomedicine, Università Politecnica delle Marche Ancona, Italy; ^3^Center for Celiac Research, MassGeneral Hospital for Children and the Celiac Program, Harvard Medical School Boston, MA, United States

**Keywords:** gluten sensitivity, gluten, gluten-free diet, challenge, non-celiac, placebo

## Abstract

**Background:** Non-celiac gluten sensitivity (NCGS) is a clinical entity characterized by intestinal and/or extra-intestinal symptoms related to the ingestion of gluten in individuals that are not affected by either celiac disease (CD) or wheat allergy (WA). Since we do not have specific biomarkers for NCGS, the diagnosis is based on the evidence of a clear relationship between the ingestion of gluten (re-challenge) and clinical symptoms, after a remission during the gluten-free diet (GFD). Several re-challenge studies have been published so far to evaluate the real prevalence of NCGS, reporting conflicting results. In the present article, we provide a systematic review with meta-analysis of the existing literature on re-challenge studies to evaluate prevalence figures of NCGS after re-challenge procedures.

**Methods:** All clinical trials performing a gluten re-challenge with or without a placebo control in patients with a suspected diagnosis of NCGS were included. Search results were limited to studies published in English language. No publication date or publication status restrictions were imposed.

**Results:** Eleven studies were included in the meta-analysis. There was a considerable heterogeneity related to different sample size, type, and amount of gluten administered, duration of challenge and different type of placebo. The overall pooled percentage of patients with a diagnosis of NCGS relapsing after a gluten challenge was 30%, ranging between 7 and 77%. The meta-analysis showed a not significant relative risk (RR) of relapse after gluten challenge as compared to placebo (RR = 0.4; 95% CI = −0.15–0.9; *p* = 0.16). The overall pooled percentage of patients with a diagnosis of NCGS relapsing after a gluten challenge performed according to the recent Salerno criteria was significantly higher as compared to the percentage of patients relapsing after placebo (40 vs. 24%; *p* = 0.003), with a significant RR of relapse after gluten challenge as compared to placebo (RR = 2.8; 95% CI = 1.5–5.5; *p* = 0.002).

**Conclusions:** The prevalence of NCGS after gluten re-challenge is low, and the percentage of relapse after a gluten or a placebo challenge is similar. However, a higher number of patients will be correctly classified with NCGS if applying the recent Salerno criteria.

## Introduction

### Rationale

Non-celiac gluten sensitivity (NCGS) is a syndrome characterized by intestinal and/or extra-intestinal symptoms related to the ingestion of gluten in individuals that are not affected by either celiac disease (CD) or wheat allergy (WA) (Sapone et al., 2012; Catassi et al., 2013; Schuppan et al., 2015). The clinical presentation of NCGS is a combination of gastro-intestinal symptoms (i.e., abdominal pain, bloating, diarrhea, or constipation), and systemic manifestations, including disorders of the neuropsychiatric area such as “foggy mind,” depression, headache, and fatigue (Catassi, 2015). The first cases of NCGS were described in the 1970s (Ellis and Linaker, 1978; Cooper et al., 1981) but did not receive much recognition from clinicians until the twenty-first century. In 2010 Sapone et al. rediscovered this condition describing its clinical and pathophysiological features (Sapone et al., 2010). Since then, the number of persons treated with the gluten-free diet (GFD) because of a wide range of symptoms has exponentially increased, as well as the number of papers reporting on NCGS. However, the real prevalence of NCGS is not clearly defined yet (Catassi, 2015). Recent surveys report about 10% of adults currently starting a GFD for different reasons, but many of these cases are self-diagnoses not verified by a doctor (Aziz et al., 2014). Indirect evidence suggests that “true” NCGS is around 1% (Volta et al., 2014; Catassi, 2015). Since we still do not have specific biomarkers for NCGS, the diagnosis is based on the evidence of a clear relationship between the ingestion of gluten and clinical symptoms. The aim of the diagnostic work-up should be two-fold: (1) assessing the clinical response to the GFD and (2) verifying the effect of reintroducing gluten after a period of treatment with the GFD (i.e., re-challenge; Catassi, 2015). The standardization of the diagnostic protocol has been recently published by a group of experts on gluten-related disorders, and is called as “Salerno criteria” (Catassi et al., 2015). First of all, the patient should exhibit at least a 30% decrease in gastrointestinal and/or extra-intestinal symptoms after a 6-week GFD. Then, a double-blind placebo controlled (DBPC) re-challenge with crossover should be performed, with 1 week of duration for each challenge and the wash-out period in between. The recommended daily doses for gluten are 8 g, whereas the placebo should be gluten-free (Catassi et al., 2015).

Several re-challenge studies have been published so far to evaluate the “real” prevalence of NCGS, reporting conflicting results.

### Objectives and research question

In the present article, we provide a systematic review and meta-analysis of the existing literature on re-challenge studies in patients diagnosed with NCGS with the aim of: (a) evaluate prevalence figures of NCGS after re-challenge procedures; (b) try to answer the question of whether there is evidence of a causal relationship between gluten and relapsing symptoms as compared to a placebo effect.

## Methods

### Protocol

Before review and meta-analysis, we developed a protocol, including eligibility criteria, search strategies, criteria for study selection, methods for extracting related data, and methods for assessing study quality and statistical methodology, according to the Preferred Reporting Items for Systematic Reviews and Meta-Analyses (PRISMA) guidelines (Moher et al., 2009).

### Eligibility criteria

All clinical trials performing a gluten re-challenge with or without a placebo control in patients with a suspected diagnosis of NCGS were considered for inclusion in this meta-analysis. Patients with NCGS were the focus of our search. NCGS was defined as self-reported gluten intolerance resulting in gastrointestinal and/or extra-intestinal symptoms, which remitted upon gluten withdrawal, with documented exclusion of WA (IgE skin testing) and CD (sero-negativity and absence of villous atrophy; Catassi et al., 2013, 2015). Studies on patients suffering from CD or other gluten-related disorders (gluten-ataxia, autism, etc.) or WA were excluded. Search results were limited to studies published in English language. No publication date or publication status restrictions were imposed. Our outcome measures were: (1) the incidence of relapse after re-challenge with gluten in patients with a diagnosis of NCGS; (2) the incidence of relapse with gluten as compared to placebo after a DBPC re-challenge; (3) the incidence of relapse with gluten as compared to placebo after a DBPC re-challenge performed as recommended by Salerno criteria; (4) meta-correlation between relapse after a DBPC re-challenge and the amount of gluten administered together with the duration of challenge.

### Information sources

Studies were identified by searching electronic databases and scanning reference list of articles (the reference lists of articles were reviewed to include further appropriate articles), and by consultation with experts in the field.

### Search

This search was applied to the Medline database using PubMed by search terms as “non celiac OR nonceliac OR non-celiac OR noncoeliac AND gluten sensitivity.” All studies described in this meta-analysis were published between 2011 and 2016. The last search was run on November 2016.

### Study selection

Eligibility assessment was performed independently in an unblended standardized manner by three reviewers (MV, CC, and EL). We screened the retrieved records to review the full text publication. Disagreements between reviewers were resolved by consensus.

### Data collection process

We developed a data extraction sheet (based on the Cochrane Consumers and Communication Review Group's data extraction template), pilot tested it on three randomly selected studies, and refined it accordingly. Two review authors extracted the following data from included studies and the third author checked the extracted data.

### Data items

Information was extracted from each included study on: (1) characteristics of study participants (including number and age); (2) the type of study and how it was performed (study design, number of patients exposed to gluten and placebo, type of symptoms before the challenge, duration of GFD, amount of gluten administered, type of gluten and duration of gluten challenge, type of placebo); (3) type of outcome measure (number of patients relapsing on gluten, number of patients relapsing on placebo, type of symptoms during the relapse).

### Summary measures

We conducted different meta-analysis with the random effect mode using the metafor package of the R system. We performed the first meta-analysis to assess the incidence of relapse of NCGS after re-challenge with gluten in patients with a diagnosis of NCGS. We used as effect-size the Incidence Rate Ratio (IRR) since for each group of patients we had the person-times with weeks as units of time. With a second meta-analysis we analyzed the relative risk (RR) and the IRR of relapse in two classes of patients: those receiving gluten as compared to those receiving placebo. We performed a third meta-analysis including only studies in which the gluten re-challenge was performed accordingly to Salerno criteria (i.e., challenge with ≥ 8 g of gluten/day; duration of challenge ≥ 1 week; Catassi et al., 2015) and we analyzed the RR and the IRR in the two classes of patients (gluten vs. placebo). Finally, a meta-analysis using as effect-size the correlation among the percentage of the patients relapsing, the amount of gluten administered in each study and the time window of the challenge has been conducted. The Mantel-Haenszel inverse variance was used for pooling (Fleiss, 1993).

### Planned method of analysis

As a measure of heterogeneity, we computed the statistic I2, defined as the percentage of total variance across studies attributable to heterogeneity rather than chance.

### Risk of bias across studies

To ascertain the validity of the eligible studies, the study design, the size and representativeness of the study population, the validity of outcomes, and the quality of the statistical analysis were taken into account. We assessed the methodological quality of included studies in accordance with the guidelines of the Cochrane Consumers and Communication Review Group. In all cases, three authors (MV, CC, and EL) assessed the quality of the studies included, with any disagreement resolved by discussion and consensus.

## Results

### Study characteristics

The characteristics of the 11 studies included in the meta-analysis are summarized in Table 1 (Biesiekierski et al., 2011, 2013; Carroccio et al., 2012; Brottveit et al., 2013; Capannolo et al., 2015; Di Sabatino et al., 2015; Shahbazkhani et al., 2015; Zanini et al., 2015; Elli et al., 2016; Picarelli et al., 2016; Rosinach et al., 2016) 5 studies were double-blind randomized placebo-controlled clinical (RDBPC) studies with a cross-over design (Carroccio et al., 2012; Biesiekierski et al., 2013; Di Sabatino et al., 2015; Zanini et al., 2015; Elli et al., 2016) but one of them did not report information on the number of patients relapsing after placebo (Carroccio et al., 2012); four were RDBPC studies without a cross-over design (Biesiekierski et al., 2011; Shahbazkhani et al., 2015; Picarelli et al., 2016; Rosinach et al., 2016) and two were open studies without a placebo control (Brottveit et al., 2013; Capannolo et al., 2015). Only four studies performed the gluten challenge as recommended by Salerno criteria: they were all RDBPC studies without a cross-over design (Biesiekierski et al., 2011; Shahbazkhani et al., 2015; Rosinach et al., 2016) and one RDBPC study with cross-over (the one not reporting information on the number of patients relapsing after placebo; Carroccio et al., 2012).

**Table 1:** Characteristics of studies included in the review.

**Reference and type of study**	**n patients**	**Symptoms pre**	**GFD**	**Challenge**	**Gluten (*g*)**	**Gluten vehicle**	**Symptoms post**
(Biesiekierski et al., 2011)	34	IBS	6 w	6 w	16	muffin	GI + extra GI
DBRPCT according Salerno's criteria							
(Carroccio et al., 2012)	920	NCGS	4 w	2 w	20	cps	GI
DBRPCT with cross-over							
(Biesiekierski et al., 2013)	37	NCGS	6 w	3 d	16	meal	GI + fatigue
DBRPCT with cross-over							
(Brottveit et al., 2013)	22	NCGS	variable	3 d	4.4	bread	GI
Open trial							
(Shahbazkhani et al., 2015)	72	IBS	6 w	6 w	52	flour	GI + fatigue
DBRPCT according Salerno's criteria							
(Di Sabatino et al., 2015)	59	NCGS	11 m	1 w	4.4	cps	GI + extra GI
DBRPCT with cross-over							
(Zanini et al., 2015)	35	NCGS	6 m	10 d	7.9	flour	GI + extra GI
DBRPCT with cross-over							
(Capannolo et al., 2015)	364	NCGS	6 m	1 m	ns	ns	GI + extra GI
Open trial							
(Elli et al., 2016)	97	IBS	3 w	1 w	5.6	cps	GI
DBRPCT with cross-over							
(Picarelli et al., 2016)	26	NCGS	1 w	1 d	10	cookie	GI + extra GI
DBRPCT							
(Rosinach et al., 2016)	18	NCGS	12 m	6 m	16.2	flour	GI
DBRPCT according Salerno's criteria							

DBRPCT, double-blind randomized placebo controlled trial; IBS, irritable bowel syndrome; w, weeks; cps, capsule; ns, not specified; GI, gastro-intestinal

All the 11 studies were included in the first meta-analysis (Biesiekierski et al., 2011, 2013; Carroccio et al., 2012; Brottveit et al., 2013; Capannolo et al., 2015; Di Sabatino et al., 2015; Shahbazkhani et al., 2015; Zanini et al., 2015; Elli et al., 2016; Picarelli et al., 2016; Rosinach et al., 2016). Eight RDBPC studies were included in the second meta-analysis (Biesiekierski et al., 2011, 2013; Di Sabatino et al., 2015; Shahbazkhani et al., 2015; Zanini et al., 2015; Elli et al., 2016; Picarelli et al., 2016; Rosinach et al., 2016; open studies Brottveit et al., 2013; Capannolo et al., 2015) and the one RDBC not reporting information on the number of patients relapsing after placebo (Carroccio et al., 2012) were excluded. Three studies (Biesiekierski et al., 2011; Shahbazkhani et al., 2015; Rosinach et al., 2016) performing the gluten challenge as recommended by Salerno criteria were included in the third meta-analysis the one not reporting information on the number of patients relapsing after placebo (Carroccio et al., 2012) was excluded. Eight RDBPC studies (Biesiekierski et al., 2011, 2013; Di Sabatino et al., 2015; Shahbazkhani et al., 2015; Zanini et al., 2015; Elli et al., 2016; Picarelli et al., 2016; Rosinach et al., 2016) were included in the fourth meta-analysis (open studies and the one RDBC not reporting information on the number of patients relapsing after placebo (Carroccio et al., 2012) were excluded).

The 11 included studies involved 1,684 participants. The primary inclusion criteria entailed patients (median age 50 years, range: 17–78) with a diagnosis of NCGS. None of the articles reported a power calculation to determine the population size necessary to answer the research question. The sample size was highly different across the study, ranging from 18 to 920 patients enrolled.

All of the studies generally included patients with intestinal bowel syndrome (IBS) like symptoms. Few studies included patients with also extra-intestinal symptoms (such as, fatigue, headache, numbness, mental confusion, anxiety/depression, and fibromyalgia-like symptoms). The type of gluten administered, the quantity of gluten and the duration of challenge were highly different among the studies, as shown in Table 1. All research groups used different placebo components, including gluten-free muffins and bread, xylose, whey protein or starch. Of note, two studies used Fermentable Oligosaccharides, Di-, Monosaccharides and Polyols (FODMAP)—containing placebo, like corn starch (Shahbazkhani et al., 2015) and corn starch, lactose, and fructans (Zanini et al., 2015).

### Synthesized findings

#### Incidence of relapse after gluten challenge

**Figure 1:**
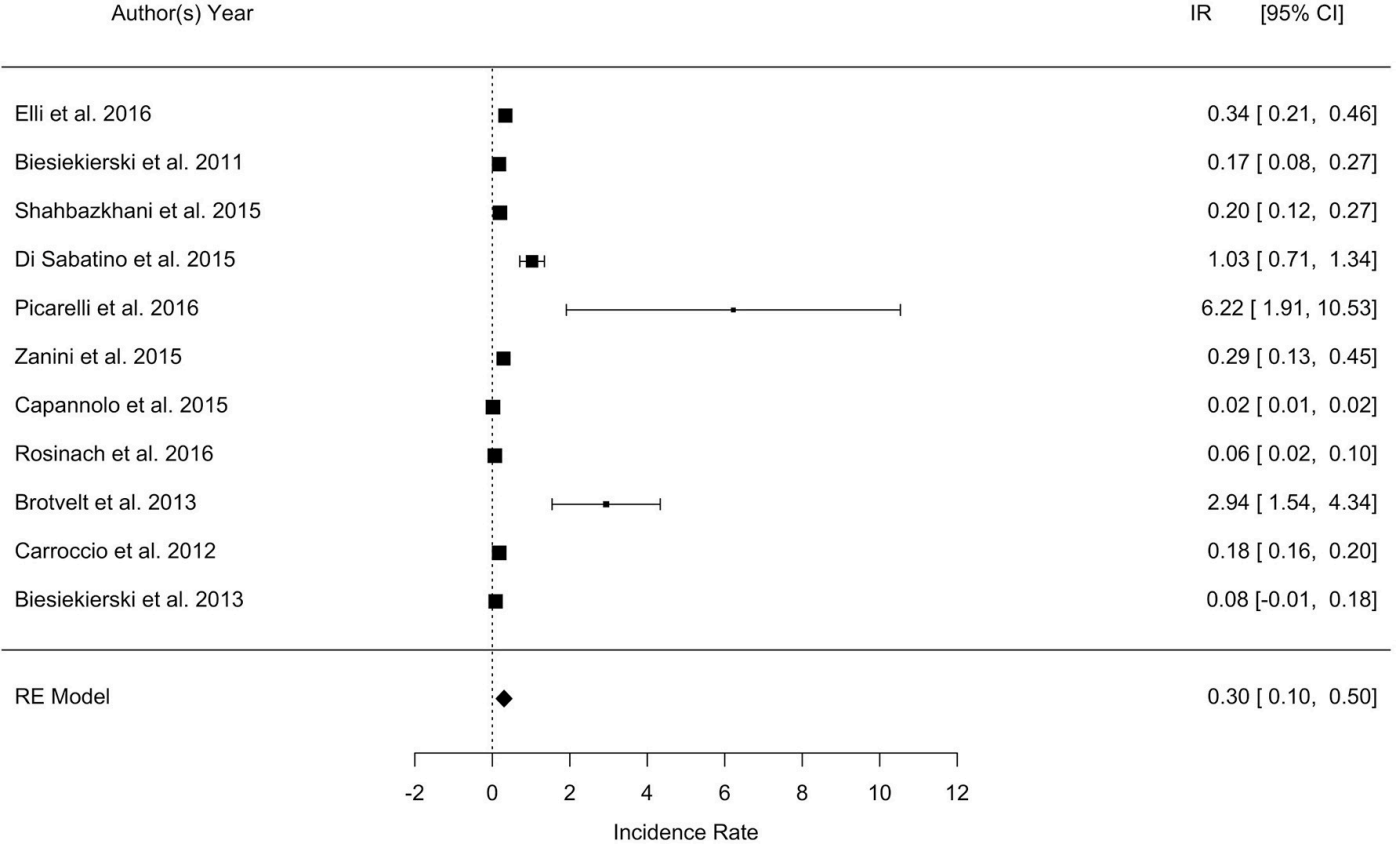
Forest plot showing the Incidence Rate (IR) of relapsing after a gluten challenge in patients with a diagnosis of non-celiac gluten sensitivity.

The overall pooled percentage of patients with a diagnosis of NCGS relapsing after a gluten challenge was 30%, with a wide range across the studies varying between 7 and 77%. The meta-analysis showed that the IRR of relapse after gluten challenge in patients with a diagnosis of NCGS is 0.3 (95% CI, 0.1–0.5; *p* = 0.003; I2 = 99.5%). The forest plot is reported in Figure 1. The considerable heterogeneity across the study was related to different sample size, different type and amount of gluten administered, and different duration of challenge.

The results of only RDBPC studies (with or without a cross-over design) showed a percentage of relapse after gluten challenge ranging from 8 to 57% (Biesiekierski et al., 2011, 2013; Carroccio et al., 2012; Di Sabatino et al., 2015; Shahbazkhani et al., 2015; Zanini et al., 2015; Elli et al., 2016; Picarelli et al., 2016; Rosinach et al., 2016) The amount of gluten administered was ≥ 8 g/die in all studies except three (Di Sabatino et al., 2015; Zanini et al., 2015; Elli et al., 2016); the duration of challenge was ≥ 1 week in all, except one (Picarelli et al., 2016). Symptoms evaluated after challenge were prevalently IBS-like. Among the open trials, the first by Brottveit et al. showed that 17/22 (77%) patients suffering from IBS-like symptoms relapsed after a 3-days gluten challenge with 4.4. g of gluten (Brottveit et al., 2013), while Capannolo et al. found that only 27/364 (7%) patients had a relapse of either gastro-intestinal or neurological symptoms after a gluten challenge for 1 month with a non specified amount of gluten (Capannolo et al., 2015).

#### Incidence of relapse after gluten challenge as compared to placebo

When comparing the relapse between patients receiving gluten with respect to those receiving placebo, RDBPC studies with or without a cross-over design found a similar percentage of relapse after either a gluten or a placebo challenge (RDBPC with cross-over: 34 vs. 32%, respectively; *p* = 0.7. RDBPC without cross-over: 38 vs. 28%, respectively; *p* = 0.2; Biesiekierski et al., 2011, 2013; Di Sabatino et al., 2015; Shahbazkhani et al., 2015; Zanini et al., 2015; Elli et al., 2016; Picarelli et al., 2016; Rosinach et al., 2016).

**Figure 2:**
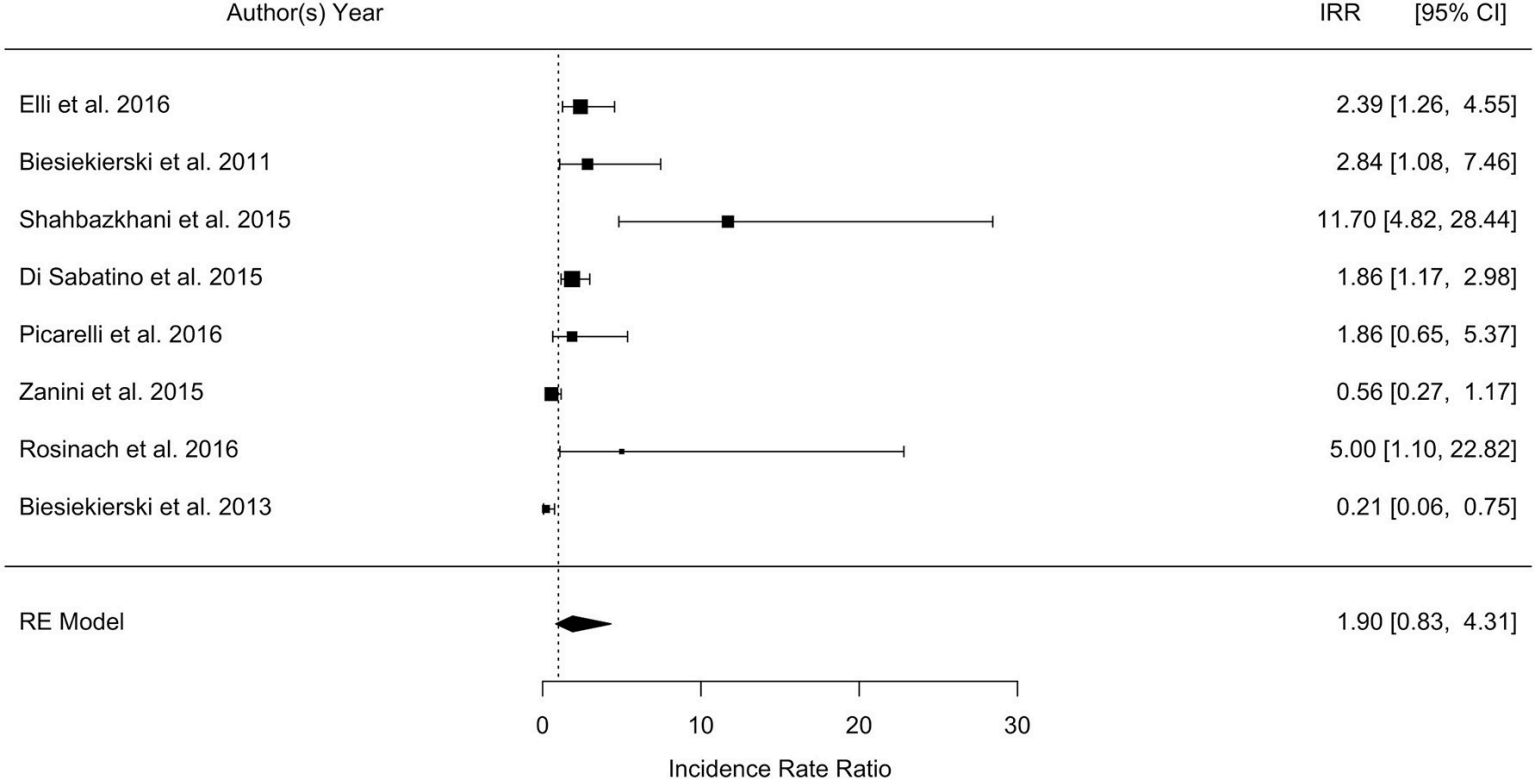
Forest plot showing the Incidence Rate Ratio (IRR) of relapsing after a gluten challenge as compared to placebo in patients with a diagnosis of non-celiac gluten sensitivity.

The overall pooled percentage of patients with a diagnosis of NCGS relapsing after a gluten challenge was 36% as compared to 31% relapsing after placebo (*p* = 0.2). The meta-analysis showed a RR of relapse after gluten challenge as compared to placebo of 0.4 (95% CI, −0.15 −0.9; *p* = 0.16; I2 = 80.3%), and a IRR of 0.6 (95% CI, −0.2 −1.5; *p* = 0.13; I2 = 87%), as shown in Figure 2. The considerable heterogeneity across the study was related to different sample size, type and amount of gluten administered, duration of challenge, and different type of placebo.

#### Incidence of relapse after gluten challenge as compared to placebo according to the salerno criteria

**Figure 3:**
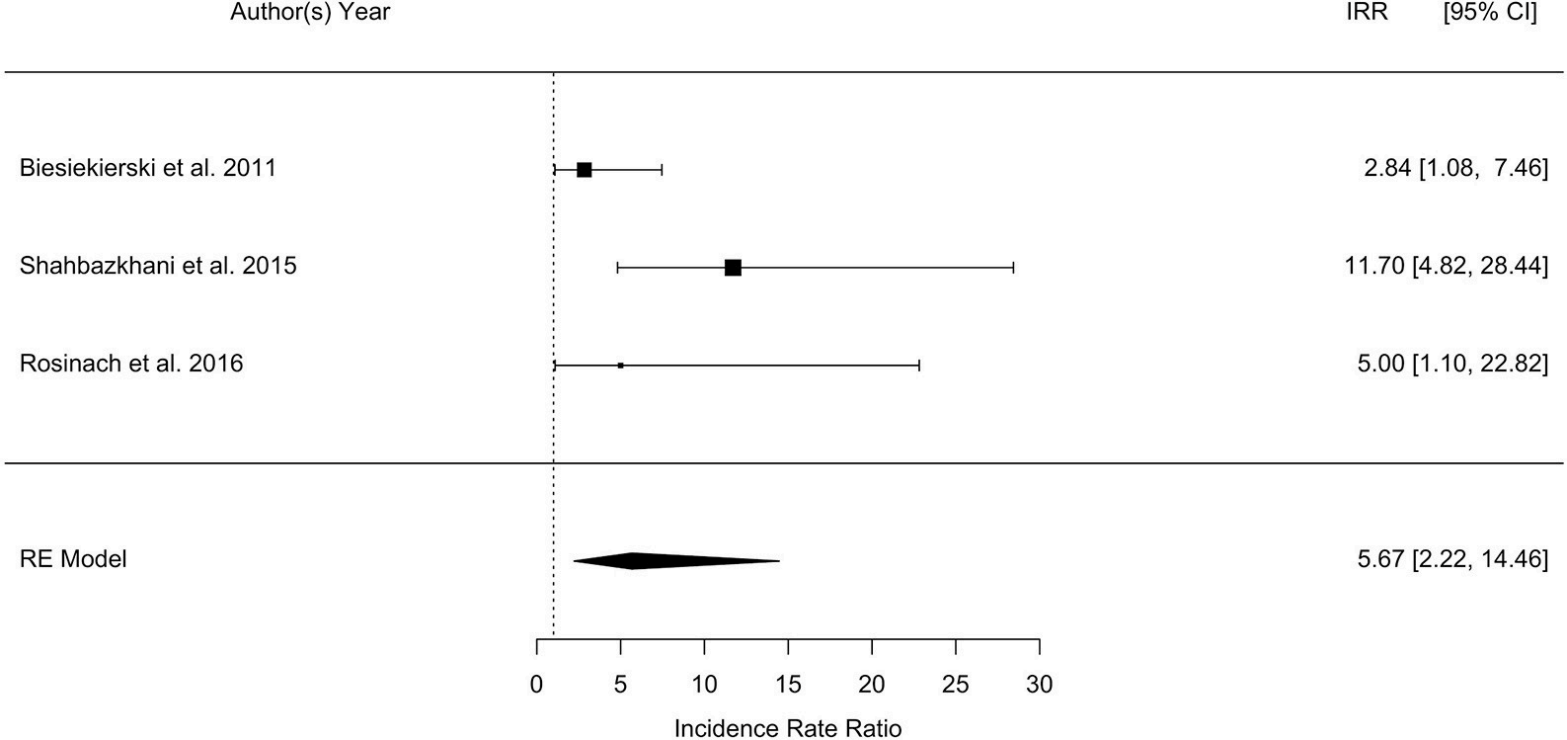
Forest plot showing the Incidence Rate Ratio (IRR) of relapsing after a gluten challenge performed as recommended by the Salerno criteria as compared to placebo in patients with a diagnosis of non-celiac gluten sensitivity.

The overall pooled percentage of patients with a diagnosis of NCGS relapsing after a gluten challenge performed according to the Salerno criteria was significantly higher as compared to the percentage of patients relapsing after placebo (40 vs. 24%; *p* = 0.003; Biesiekierski et al., 2011; Shahbazkhani et al., 2015; Rosinach et al., 2016). The meta-analysis showed a highly significant RR of relapse after gluten challenge as compared to placebo of 2.8 (95% CI, 1.5–5.5; *p* = 0.002; I2 = 80.3%), and a highly significant IRR of 5.7 (95% CI, 2.2–14.5; *p* = 0.0003; I2 = 87%). The forest plot showing IRR is reported in Figure 3.

#### Meta-correlation analysis

**Figure 4:**
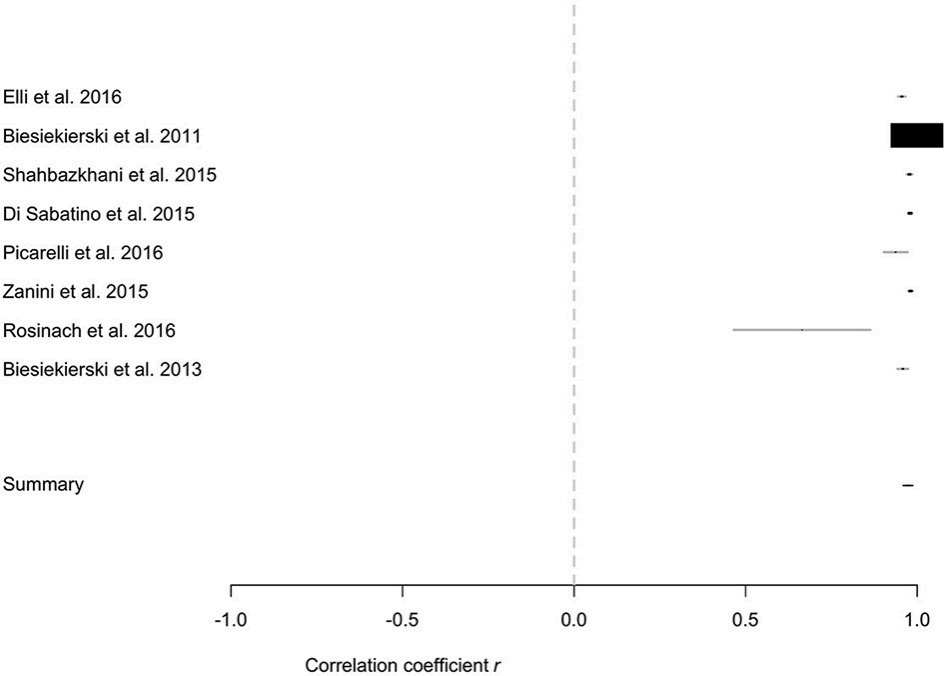
Meta-correlation between the percentage of relapse and the percentage of the patients enrolled, the amount of gluten administered in each study and the time window of the challenge.

Finally, the meta-analysis using as effect-size the correlation among the percentage of the patients relapsing, the amount of gluten administered in each study and the time window of the challenge showed that the percentage of relapse was highly correlated with the amount of gluten and the duration of challenge (Figure 4).

## Discussion

### Summary of main findings

The present meta-analysis shows that the prevalence of NCGS after gluten re-challenge in patients with a suspected diagnosis of NCGS is low, and the percentage of relapse after either a gluten or a placebo challenge is similar. However, when the gluten re-challenge procedure was performed according to the recommended Salerno criteria, the percentage of relapse after a gluten challenge is significantly higher compared to placebo. Therefore, a higher number of patients would have been correctly classified with NCGS by applying the recent Salerno criteria.

Clinical trials performed so far to rigorously investigate the NCGS are still in their infancy. To date, only a few prospective randomized clinical trials on the role of gluten in inducing symptoms in individuals without CD have been published, each with its own strengths and limitations. This has resulted in a significant and perhaps undue degree of skepticism regarding the nature and the existence of this condition (Lebwohl and Leffler, 2015). However, an increasing number of persons claim to suffer from NCGS, and physicians are called to scientifically define the condition. In view of the “gluten free fad” we have to distinguish mere claims of a health benefit of GFD from real disease, and rule out several other differential diagnoses before label a patient with NCGS (Schuppan et al., 2015).

The definition of NCGS has been recently discussed at three consensus conferences, leading to three different publications (Sapone et al., 2012; Catassi et al., 2013; Ludvigsson et al., 2013). Given the uncertainties about this disease and the lack of specific biomarkers, according to these consensus conferences NCGS should be defined by the following criteria: a clinical entity induced by the ingestion of gluten leading to intestinal and/or extra-intestinal symptoms that resolve on GFD, when CD and WA have been ruled out. However, one of the most controversial issue is the real role of gluten in causing symptoms in NCGS patients (Fasano et al., 2015).

The present meta-analysis of re-challenge studies suggests that a high proportion of patients suspected to have a NCGS cannot reach a formal diagnosis of NCGS after a gluten challenge. Indeed, the IRR of relapse after an open challenge with gluten was 0.3, and the percentage of patients relapsing after a gluten challenge was 36% as compared to 31% relapsing after placebo, with a not significant RR and IRR of relapse after gluten challenge as compared to placebo. These findings may be explained by the following: (a) the symptoms experienced in normal life by patients labeled as NCGS when consuming gluten may be related to a psychological anticipation of intolerance, that seems to be a “nocebo” effect; commercial pressure and emotional factors may be important, even in individuals with low levels of somatization (Zanini et al., 2015; Molina-Infante and Carroccio, 2017); (b) mainly with respect to gastro-intestinal symptoms, there may be a significant overlap with IBS, intolerance to FODMAPs, WA (which is frequently missed with conventional blood IgE and skin test), or small bacterial overgrowth (Schuppan et al., 2015; Molina-Infante and Carroccio, 2017); (c) since NCGS may be a transient disorder, some patients could have undergo the re-challenge at the time when their gluten sensitivity could have overcome; and finally, (d) methodological issues in the gluten challenge procedure of the majority of studies performed so far may have affected the results. Indeed, as shown by the high heterogeneity across the studies, the duration of gluten challenge, the amount of gluten administered, the type of placebo, and the type of symptoms recorded were significantly different. As for the duration of gluten challenge, it ranged between 1 day and 6 months, with some studies evaluating the relapse only after 1 or 3 days of challenge, that might be a time too short for detecting mild or fluctuating symptoms. Also the type and the amount of gluten may be an issue: the amount ranged between 4.4 and 52 g or it was not specified. A dose of 5 g of gluten is far from the average daily intake of gluten in developed countries (10–15 g; Van Overbeek et al., 1997), and the diagnostic yield of the gluten challenge may be increased only with higher gluten doses. As far as the gluten “vehicle,” several studies used gelatin capsules, that are strongly discouraged (Catassi et al., 2013). The best-suited vehicle, yet to be developed, could take form of a muesli bar, bread, or muffin, and should contain homogeneously distributed cooked gluten (Catassi, 2015). The placebo substances may be also a concern; they must be completely gluten-free, (Catassi et al., 2013) but two studies used FODMAP-containing corn starch (Di Sabatino et al., 2015; Shahbazkhani et al., 2015). Symptoms related to this placebo substance might have been misinterpreted as nocebo effect and potentially prevented a diagnosis of NCGS (Molina-Infante and Carroccio, 2017). Finally, the type of symptoms recorded before and after the re-challenge were different across the studies, some of that including both intestinal and extra-intestinal manifestations and other evaluating only IBS-like complaints. For the experienced clinician, extra-intestinal symptoms serve as the best indicator of the disease, and are increasingly recognized as hallmarks of NCGS (Schuppan et al., 2015). However, a quantitative analysis of the number of patients with relapse of gastro-intestinal as compared to extra-intestinal symptoms can't be performed so far from available studies, because only few studies reported the type of symptoms relapsing.

Noteworthy, when the gluten re-challenge was performed according to the current recommended procedure (i.e., the Salerno criteria) the overall percentage of patients relapsing after gluten was significantly higher as compared to placebo (40 vs. 24%; *p* = 0.003), and the meta-analysis showed a highly significant RR and IRR of relapse after gluten as compared to placebo. This finding supports the existence of a causal relationship between gluten and relapsing symptoms, and strongly suggest to follow the Salerno expert recommendations when suspecting the diagnosis of NCGS, until a valid biomarker will be available, as previously reported (Skodje et al., 2017). This is further supported by the results of the meta-correlation analysis showing that the percentage of relapse was highly correlated with the amount of gluten and the duration of challenge.

It has been recently hypothesized that besides gluten, other components of gluten-containing cereals may be the trigger/s of the immunologic events leading to NCGS. Gluten peptides different from those that activate T cells in CD patients could trigger innate immune responses in NCGS, but evidence for their role *in vivo* has been lacking (Fasano et al., 2015). Recently, *in vitro* and *in vivo* studies suggest that wheat Amylase-Trypsin Inhibitors (ATIs) could play a major role as triggers of the innate immune response in intestinal monocytes, macrophages, and dendritic cells eventually leading to NCGS (Catassi et al., 2013; Schuppan and Zevallos, 2015). ATIs are plant-derived proteins that inhibit enzymes produced by common parasites, such as, mealworms and meal bugs, in wheat (Schuppan and Zevallos, 2015). Importantly, the innate immune response elicited is mainly confined to ATIs of gluten containing grains. Thus, a GFD is also essentially ATI-free (Schuppan and Zevallos, 2015). Therefore, it would be essential when performing a gluten challenge that the gluten administered is prepared/tested for ATI bioactivity to contain at least 0.3 g of ATIs/8 g of gluten or gluten should be used with defined ATI content (Catassi et al., 2015). This has never been performed so far.

## Conclusions

In conclusion, the meta-analysis of the existing literature on re-challenge studies in patients diagnosed with NCGS shows that the prevalence of confirmed NCGS after re-challenge test is low. However, a causal relationship between gluten and relapsing symptoms is observed in 40% of patients when performing a gluten challenge according the Salerno criteria. This gluten challenge procedure is highly recommended until a valid biomarker will be available.

Further studies are needed to: (1) identify specific biomarkers, that would aid in positively diagnosing patients with NCGS, e.g., after a short-term challenge, and (2) perform gluten/ATI challenge in well-defined patients to evaluate the pathophysiological role of gluten as compared to ATI in triggering the disease.

## Author contributions

EL had substantial contribution to the conception of the work, the acquisition and interpretation of data, wrote the work, final approved the version to be published, and agreed to be accountable for all aspects of the work. AP had substantial contribution to the analysis of data, wrote the work, final approved the version to be published, and agreed to be accountable for all aspects of the work. MV, GC, SG, and AV had substantial contribution to the acquisition of data, drafted the work, final approved the version to be published, and agreed to be accountable for all aspects of the work. CC had substantial contribution to the conception of the work, revised it critically for important intellectual content, final approved the version to be published, and agreed to be accountable for all aspects of the work.

## Conflict of interest statement

EL served as consultant for Dr. Schar. CC served as consultant for Dr. Schr. The other authors declare that the research was conducted in the absence of any commercial or financial relationships that could be construed as a potential conflict of interest. The reviewer FG and handling Editor declared their shared affiliation and the handling Editor states that the process met the standards of a fair and objective review.
